# *Col11a1a* Expression Is Required for Zebrafish Development

**DOI:** 10.3390/jdb8030016

**Published:** 2020-08-28

**Authors:** Makenna J. Hardy, Jonathon C. Reeck, Ming Fang, Jason S. Adams, Julia Thom Oxford

**Affiliations:** 1 Biomolecular Research Center; Boise State University, Boise, ID 83725, USA; makennahardy@u.boisestate.edu (M.J.H.); jonathonreeck@boisestate.edu or or ming.fang@beigene.com (M.F.); 2 Biomolecular Sciences Graduate Program; Boise State University, Boise, ID 83725, USA; 3 Department of Biological Sciences, Boise State University, Boise, ID 83725, USA; 4 Department of Physiology and Developmental Biology, Brigham Young University, Provo, UT 84602, USA; Jason_Adams@byu.edu; 5 Center of Biomedical Research Excellence in Matrix Biology, Boise State University, Boise, ID 83725, USA

**Keywords:** collagen, *Col11a1a*, alternative splicing, minor fibrillar collagen, zebrafish, Stickler type 2 syndrome, Marshall syndrome, fibrochondrogenesis

## Abstract

The autosomal dominant chondrodystrophies, the Stickler type 2 and Marshall syndromes, are characterized by facial abnormalities, vision deficits, hearing loss, and articular joint issues resulting from mutations in *COL11A1*. Zebrafish carry two copies of the *Col11a1* gene, designated *Col11a1a* and *Col11a1b*. *Col11a1a* is located on zebrafish chromosome 24 and *Col11a1b* is located on zebrafish chromosome 2. Expression patterns are distinct for *Col11a1a* and *Col11a1b* and *Col11a1a* is most similar to *COL11A1* that is responsible for human autosomal chondrodystrophies and the gene responsible for changes in the chondrodystrophic mouse model *cho/cho*. We investigated the function of *Col11a1a* in craniofacial and axial skeletal development in zebrafish using a knockdown approach. Knockdown revealed abnormalities in Meckel’s cartilage, the otoliths, and overall body length. Similar phenotypes were observed using a CRISPR/Cas9 gene-editing approach, although the CRISPR/Cas9 effect was more severe compared to the transient effect of the antisense morpholino oligonucleotide treatment. The results of this study provide evidence that the zebrafish gene for *Col11a1a* is required for normal development and has similar functions to the mammalian *COL11A1* gene. Due to its transparency, external fertilization, the *Col11a1a* knockdown, and knockout zebrafish model systems can, therefore, contribute to filling the gap in knowledge about early events during vertebrate skeletal development that are not as tenable in mammalian model systems and help us understand *Col11a1*-related early developmental events.

## 1. Introduction

The molecular mechanisms directing developmental patterning and gene expression at early stages in vertebrate development are conserved in many respects between zebrafish and humans, with cartilage forming the majority of the vertebrate embryonic skeleton in early development, relying on mesenchymal cell proliferation and condensation [1,2]. Chondroprogenitor proliferation and terminal differentiation lead to the formation of precisely sized and shaped skeletal elements [3]. Cartilage defects during this process can lead to chondrodystrophies that may include abnormal bone formation, joint dysfunction, and early-onset osteoarthritis [4].

In addition to skeletal symptoms, chondrodystrophies such as Stickler syndrome also includes hearing loss, and the zebrafish model system may provide insight into the mechanism that links the skeletal phenomena to hearing loss, resulting from mutations in the *Col11a1* gene. The development of the zebrafish ear is similar to other vertebrates [5,6] and zebrafish has served as a model system for the study of ear development [6,7,8].

Cartilage-related defects associated with collagen type XI have been identified in several vertebrate species including mice, humans, dogs, and zebrafish [9,10,11,12,13]. In mice, a *Col11a1* mutation causing a hereditary recessive chondrodysplasia (*cho/cho*) has provided key insight into the role of Col11a1 in the formation of cartilaginous structures. *Cho/cho* mice display severe hearing impairment due to underdevelopment of the organ of Corti in the cochlea [14] and chondrodysplasia of the limbs, palate, ribs, mandible, and trachea [15], all of which are present as transient or permanent cartilaginous structures in the developing mouse embryo. Human mutations in *COL11A1* result in similar abnormalities that constitute the Marshall and Stickler syndromes. These syndromes are similar, characterized by varying degrees of craniofacial abnormalities, such as cleft palate, myopia, retinal detachment, deafness, dental anomalies, and early-onset arthritis [16,17,18,19,20].

Collagen is the most abundant protein in connective tissue and plays an integral part in most vertebrate tissues [21]. Disturbances in cartilage collagen composition and distribution during development results in alterations in the skeletal structure that contribute to an increased risk of disease [22]. Specifically, disruption in the expression of collagen type II and XI negatively impacts the organization and complexity needed for proper functioning mature cartilage [23].

Collagen type XI belongs to the fibrillar class of collagens [24] and polymerizes with collagen type II and IX to produce heterotypic collagen fibrils [25] found in fetal and adult cartilage [26]. *Col11a1* gene expression is reported widely beyond cartilage, to include the nucleus pulposus of the intervertebral disc, the developing notochord, the vitreous humour of the mammalian eye, skeletal muscle, brain tissue, tendons, heart valves, skin, the tectorial membrane of the inner ear, intestinal epithelia and smooth muscle of the intestine, the calvaria, and endochondral bones [26,27,28].

In our previous study, we identified zebrafish orthologues of the minor fibrillar collagen genes and analyzed the exons included within the specific splice forms. We characterized the temporal and spatial expression patterns of the *Col11a1a* splice-forms in the developing zebrafish embryo and found these splice forms to be prevalent in the ear, notochord, and Meckel’s cartilage.

In this study, we designed antisense morpholino oligonucleotides (AMOs) that effectively block translational initiation as well as intron/exon splicing of exon 6a within the alternatively spliced variable region. The results that we present here from this knockdown technique using AMOs further substantiate the identification of the homologous zebrafish genes as orthologues of *COL11A1*. The result of the exclusion of exon 6a also resulted in malformations in the otoliths in the ear, of the notochord, and of Meckel’s cartilage. Finally, results of the AMO knockdowns were compared to CRISPR/Cas9-mediated gene editing of *Col11a1a* to confirm our findings.

These investigations of zebrafish orthologues of *COL11A1* increase our understanding of the function of Col11a1 and help to establish zebrafish as a biological model for the study of collagen type XI in vertebrate development and disease.

## 2. Materials and Methods

### 2.1. Fish Maintenance, Care, and Staging

Ab/Ab *Danio rerio* embryos were obtained from Zebrafish International Resource Center (ZIRC) (Eugene, OR, USA). Juvenile and adult zebrafish were housed in an Aquatic Habitat (Apopka, FL, USA) system with regulated temperature and light cycle. Fertilized eggs were maintained in a smaller tank with a temperature of 28.5 °C, 20 to 25 embryos per 100 mL. Zebrafish were euthanized with 300 mg/mL ethyl 3-aminobenzoate methane sulfonate salt (MS-222) (Sigma Aldrich, St. Louis, MO, USA), by treating for 5–10 min until the opercular movement stopped, as approved by the Boise State University Institutional Animal Care and Use Committee (AC18-014 and AC18-015). Embryos were staged before euthanization or experimentation to determine age in hours or days post-fertilization (hpf and dpf) at 28.5 °C using a Zeiss Stemi 2000-C dissecting microscope (Carl Zeiss MicroImaging, Inc., Thornwood, NY, USA).

### 2.2. PCR

Zebrafish RNA was isolated at specific developmental stages (4 h, 10 h, 24 h, 48 h, 72 h, 3.5 d, 4.5 d, and 6.5 d) and used to generate cDNAs using Retroscript (Ambion, Austin, TX, USA). cDNA was used as a template in PCR reactions with primers flanking the variable region of the *Col11a1a* chain. Ten picomoles of each primer and 2 µL of cDNA were added to 22 µL of PCR master mix generated by adding water to Ready-To-Go PCR Beads (Amersham Biosciences, Piscataway, NJ, USA). The final reaction (25 µL) contained 1.5 U Taq DNA Polymerase, 10 mM Tris-HCl, pH 9.0 at room temperature, 50 mM KCl, 1.5 mM MgCl_2_, 200 µM of each dNTP as well as bovine serum albumin (BSA). Each reaction was then incubated as follows: 95 °C for 5 min, (95 °C for 1 min, 55.5 °C for 1 min, 72 °C for 1 min) × 30 cycles, and 72 °C for 10 min. PCR products were separated by size by electrophoresis on a 2% agarose gel (Nusieve 3:1) in Tris Acetate EDTA (TAE) buffer and stained with ethidium bromide. Bands were visualized using a Kodak ID Image Station (Eastman Kodak Company, Rochester, NY, USA) trans-illuminator.

### 2.3. Cloning and Riboprobe Synthesis

Excised PCR products were purified using the Ultrafree-DA Centrifugal purification system (Millipore/Amicon, Bellerica, MA, USA) and sequenced by the Idaho State University Molecular Research Core Facility (Pocatello, ID, USA). Purified products were ligated into the PCRII vector (Invitrogen, Carlsbad, CA, USA) overnight at 14 °C. The ligation product was transformed into chemically competent TOP10 *Escherichia coli* cells (Invitrogen, Carlsbad, CA, USA). Sequence analysis was performed by the Idaho State University Molecular Research Core Facility (Pocatello, ID, USA).

Five micrograms of riboprobe-containing plasmids were linearized using 5U Hind III with 10× BSA in 20 µL total volume. Plasmid digest fragments were subsequently purified by phenol/chloroform extraction. One-tenth volume of 8 M LiCl was added to each reaction followed by the addition of 2.5 volumes of 100% ethanol. One microgram of linearized/purified plasmid was used as a template to synthesize antisense, digoxigenin-labeled probe using DIG RNA Labeling (Roche Applied Science, Indianapolis, IN, USA). A control probe was synthesized using pSPT18-neo empty plasmid. Riboprobe synthesis products were purified using P-30 Bio Spin columns (BioRad Laboratories, Hercules, CA, USA). Probe reactions were then diluted to a working concentration with hybridization buffer for in situ hybridization assays.

### 2.4. In Situ Hybridization

Zebrafish embryos were fixed in 4% paraformaldehyde in phosphate buffer (PBS) containing 8% sucrose and 0.3 μM CaCl_2_. Embryos were dehydrated, rehydrated, washed and hybridized as previously described [29]. Embryos were washed and fixed in 2% formalin in PBST overnight at 4 °C, and stored in 75% glycerol at 4 °C.

### 2.5. Antisense Morpholino Oligonucleotide Injection

Antisense morpholino oligonucleotides (AMOs) were designed to knockdown the protein expression of Col11a1a, Col11a1b, or alter specific isoforms of *Col11a1a* (Gene Tools, LLC Philomath, OR). AMOs directed to the translational start site in exon 1 of *Col11a1a* (chr24) consisted of the sequence 5′-GGGACCACCTTGGCCTCTCCATGGT-3′, and *Col11a1b* (chr2) exon 1 consisted of the sequence 5′-ACCACCTTTCCTTATCCTTATCCAT-3′ to block initiation of protein synthesis. AMOs used to block the inclusion of specific exons within the variable region were as follows:

exon 6A 5′-GTTGTGTACTGCACATAGGGAGAGG-3′;

exon 6B 5′-GTTTCACTCTCTGGAAAAAGGTTAT-3′;

exon 8 5′-CATGGCCTTATTACACCCAAAGCAA-3′.

A control AMO 5-CCTCTTACCTCAGTTACAATTTATA-3′ directed to the gene encoding β-globin of a human patient with thalassemia was used as a negative control, as this sequence should not be present in the experimental samples (Gene Tools #18633993). AMOs were injected into the yolk of one- to two-cell embryos using an Eppendorf Femtotip II microinjection needle and an injection pressure of 3.5 psi for 0.1 s with a compensation pressure of 0.22 psi (Eppendorf, Hamburg, Germany). The effectiveness of splice blocking AMOs was confirmed by RT-PCR (see Supplementary Materials). The skeletal effects of the *Col11a1* AMOs were detected by analysis of morphants directly or by using Alcian blue staining of the injected zebrafish at specific time points and compared to time-matched untreated zebrafish and those treated with the control AMO.

### 2.6. CRISPR/Cas9 Gene Editing

A CRISPR/Cas9 gene-editing approach was used to introduce a premature stop codon in *Col11a1a.* Target sequences were designed as described by Gagnon and colleagues [30]. The target sequences were identified through the CHOPCHOP webtool (https://chopchop.rc.fas.harvard.edu/). The six best targets were selected and were used to create six different guide sequences shown in Table 1. Guide sequence e201 resulted in a premature stop codon within exon 2 and was used for *Col11a1a* CRISPR/Cas9 mutant generation.

### 2.7. Statistical Analysis

One-way ANOVA was used with randomized blocking. *p*-values of <0.05 were considered statistically significant. Measurements were analyzed using SAS/STAT software, v. 9.1 (SAS System, Cambridge, MA, USA: Cytel Software Corporation, 2007).

## 3. Results

**Figure 1:**
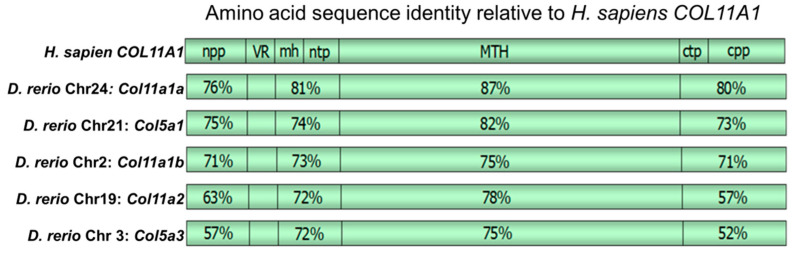
Amino acid sequence identity between *Homo sapien* and *Danio rerio* genes. Amino propeptide (npp), variable region (VR), minor helix (mh), amino telopeptide (ntp), major triple helix (MTH), carboxyl telopeptide (ctp), and carboxyl propeptide (cpp). Percentages shown indicate identity between human *COL11A1* and the zebrafish gene. Homology is observed within the npp, mh and ntp, MTH, and the ctp and cpp domains, while the degree of identity is very low for the VR. Amino acid sequence identity for other minor fibrillar collagens is shown in comparison to human *COL11A1*. The *D. rerio* chromosome 2 *Col11a1* locus identity corresponds to *Col11a1b.*

The *Danio rerio Col11a1a* gene is located on chromosome 24 (chr24) and the *Col11a1b* gene is located on chromosome 2 (chr2). Exon 1 of both *Col11a1a* and *Col11a1b* encodes the translational start site and the signal peptide, as is true for the other minor fibrillar collagen genes. Exons 2 through 5 of *Col11a1a* and *Col11a1b* encode the relatively large amino propeptide (Npp), which is also conserved for α1(XI), α2(XI), α1(V) and α3(V) alpha chains. Sequence comparison demonstrated the high degree of sequence conservation between humans and zebrafish, as shown in Figure 1. The degree of identity was used to identify the homologs of human genes within the zebrafish model system. Figure 1 demonstrates that the percent amino acid sequence identity varies among specific regions of the corresponding protein and that the most closely related zebrafish gene is that located on zebrafish chromosome 24, *Col11a1a*.

Within the npp of the amino-terminal domain, the position of four cysteines is strictly conserved, as are stretches of amino acids predicted to adopt β-strand secondary structure. Originally predicted to adopt an Ig domain fold by analysis of primary sequence [31], it has been further demonstrated that this domain is a homolog of the amino-terminal domain of thrombospondin 1 and 2 and the LNS family, so named for laminin, neurexin, and sex-hormone binding protein [32,33,34,35]. The predicted amino propeptide domain of *Col11a1a* and *Col11a1b* of zebrafish also shares sequence homology with the N-terminal domains of FACIT collagens types IX, XII, XIV, and XIX [36,37,38,39,40,41] as well as collagens type XXVIIa and XXVIIb. Divalent cation binding sites and sites of interactions with sulfated glycosaminoglycans are present in many of the LNS domains and appear to be conserved within the zebrafish genes.

In addition to the highly conserved Npp domain, within the *Col11a1a* and *Col11a1b* amino-terminal domains in zebrafish there are predicted amino acid sequences that are poorly conserved among paralogues α1(XI), α2(XI), α1(V), and α3(V) chains, and also poorly conserved among the orthologues of any one of the minor fibrillar collagens compared across species. This region is referred to as the variable region (VR) [41,42,43,44]. In zebrafish *Col11a1a* but not *Col11a1b*, the α1(XI) mRNA exists as a set of splice forms arising by the mechanism of alternative splicing [45,46]. Alternative splicing in the analogous region has also been reported for the orthologues in other vertebrate species for *Col11a1* as well as for genes encoding the α2(XI) chain. Interestingly, no alternative splicing has been reported for the alpha chains of the type V collagens. However, zebrafish may represent an exception to this rule [29,47].

We have analyzed the zebrafish intron–exon structure of the *Col11a1a* gene, comparing it to other vertebrates, and have found that a similar genomic structure exists. The *Col11a1a* on chromosome 24 gene comprises 130 kbp of genomic DNA, compared to 150 kbp in humans, with 67 exons compared to 68 exons in humans. The protein length is predicted to be slightly longer than in humans—1866 amino acids compared to 1852 amino acids. The amino acid sequence identity between zebrafish and humans, estimated by global alignment was found to be 76%, with regions of higher sequence identity in the carboxyl telopeptide and carboxyl propeptide (80%), the major triple helix (87%), and the minor helix and amino telopeptide region (81%) identity. Amino acid sequence identity for specific regions is shown in comparison to human *COL11A1* in Figure 1.

**Figure 2:**
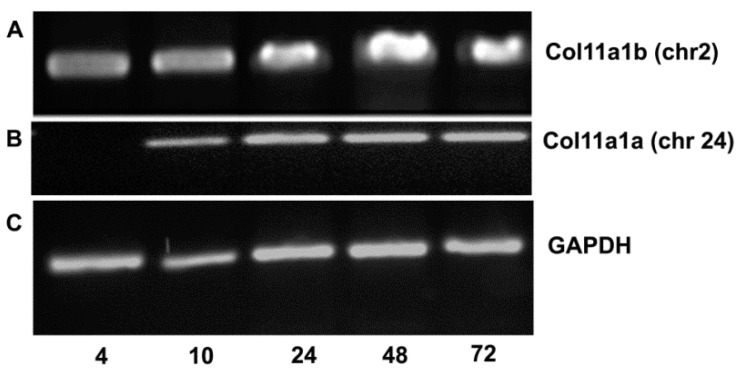
RT-PCR indicates that *Col11a1a* (chr24) is expressed between 10 and 72 hpf and *Col11a1b* (chr2) is expressed at 4 hpf through 72 hpf. (**A**) Using primers to amplify a 470 bp fragment, *Col11a1b* (*chr2*) mRNA was detected in embryos and larval fish. Time post-fertilization is indicated at the bottom of gels as 4, 10, 24, 48, and 72 hpf. (**B**) *Col11a1a* (chr24) was detected at 10 hpf through 72 hpf. (**C**) *GAPDH* was included as housekeeping gene control.

*Col11a1a* (chr24) was expressed during development, as shown in Figure 2. *Col11a1a* (chr 24) mRNA was not detectable at 4 hpf, but it was apparent at 10 hpf, during the segmentation phase of development. Expression levels were consistent through 72 hpf (Figure 2A,B). *Col11a1b* (chr2) mRNA expression was detectable at 4 hpf through the 72 hpf time point (Figure 2A). GAPDH is shown as an internal control housekeeping gene for each time point (Figure 2C).

**Figure 3:**
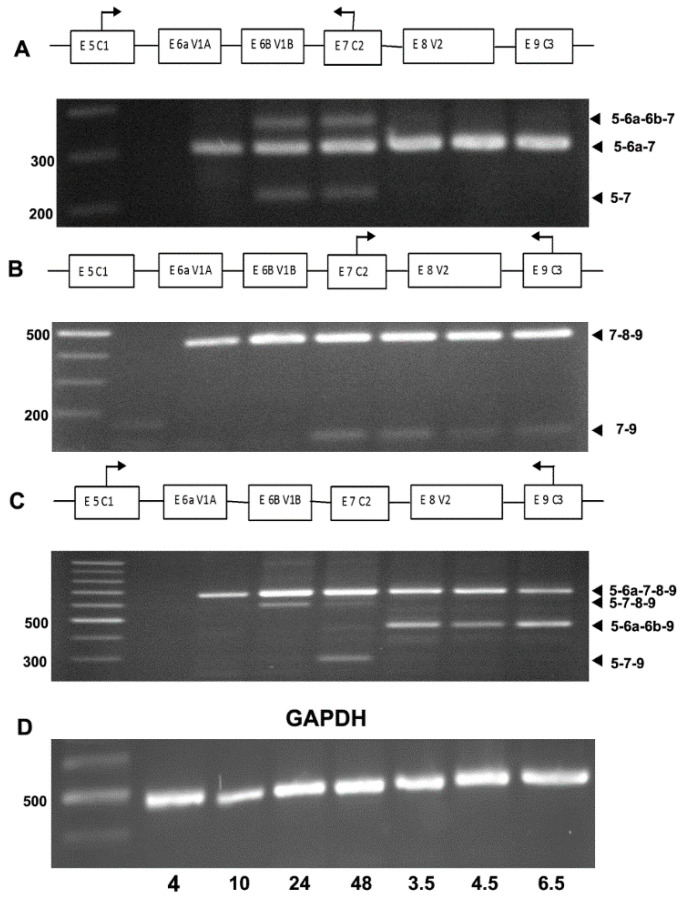
RT-PCR demonstrates alternative splicing patterns in the expression of *Col11a1a* isoforms. (**A**) PCR primers hybridizing to sequences within exons 5 and 7 were used to investigate the inclusion and exclusion of exons 6a and 6b overtime during development. Expression was detected as early as 10 hpf and continued throughout development to the last time point queried in this study, which was 6.5 dpf. (**B**) PCR primers hybridizing to sequences within exon 7 and 9 were used to investigate the inclusion and exclusion of exon 8 overtime during development. Exon 8 was included in the most predominant form of *Col11a1a* at all time points investigated. However, exon 8 was skipped in some forms of *Col11a1a*, joining exon 7 directly to exon 9, as shown by the PCR band migrating below 200 kilobases. (**C**) PCR primers hybridizing to sequences within exons 5 and 9 were used to investigate the complexity of splice form expression across the variable region of *Col11a1a* in zebrafish. The predominant splice form included exons 6a and 8, in agreement with observations shown in panels A and B. Alternative patterns of expression were observed to exclude exons 6a and 6b but include exon 8 at 24 hpf. Additionally, exclusion of exons 6a, 6b, and 8 resulted in the expression of the splice form comprising exons 5-7-9 migrating at approximately 300 kilobases at 48 hpf. (**D**) GAPDH was included as housekeeping gene control to confirm RNA content in samples representing distinct time points in development. The identity of the PCR product was verified by DNA sequencing.

Exons 6a through 8 of the variable region of *Col11a1a* were analyzed to evaluate the intron–exon boundaries across the variable region. To determine the pattern of alternative splicing that took place within this region of the zebrafish mRNA, RT-PCR was carried out using primers that would distinguish between the different possible splicing outcomes. Results indicated changes due to alternative splicing of the *Col11a1a* mRNA between 10 hpf and 6.5 dpf, as shown in Figure 3.

Alternative splicing of the *Col11a1a* (chr 24) mRNA in zebrafish generated similar splice variants to those previously been described for humans, rats, mice, and chicken with a few notable exceptions. The splice variant that includes exon 6a, 7, and 8 but excludes exons 6b (α1^6a-7-8^(XI)) was observed in zebrafish at the earliest time points. Additionally, splice variants α1^7^(XI), α1^6a-7^(XI), and α1^7-8^(XI) were also confirmed in zebrafish. Interestingly, the splice variant α1^6a-6b^(XI), was observed in zebrafish at 3.5, 4.5 and 6.5 days post-fertilization (dpf). This is noteworthy because exon 7 was thought to be constitutively expressed in all vertebrates, and exons 6a and 6b were previously thought to be either included or excluded in a mutually exclusive manner based on data from other species. This observation warrants further investigation, not only in the zebrafish system but also in humans and other vertebrate organisms. The most predominant form observed was α1^6a-7-8^(XI), which is also the predominant form found in mesenchymal stem cells in other vertebrates previously shown by our laboratory [48].

**Figure 4:**
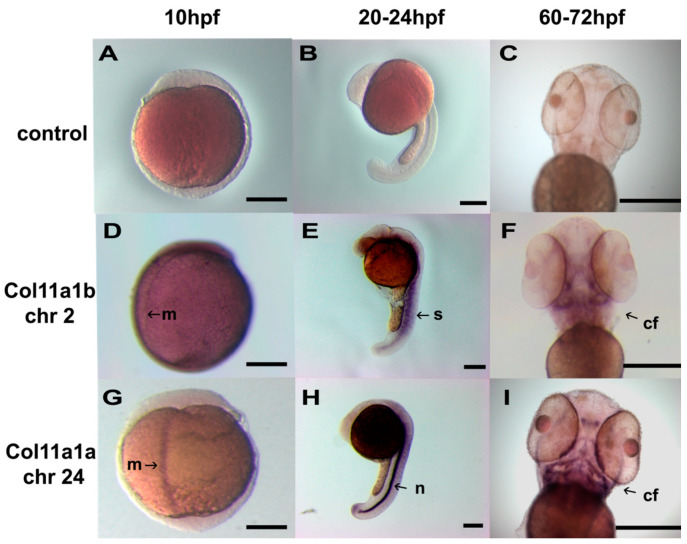
In situ hybridization of *Col11a1a* (chr 24) and *Col11a1b* (chr 2). Wild-type embryos were treated with pSPT-18 control riboprobe (**A**–**C**), *Col11a1b* (chr2) ex6-7-8-9 riboprobe (**D**–**F**), and *Col11a1a* (chr24) ex6a-7-8-9 (**G**–**I**). Embryos were observed at 10 hpf (**A**,**D**,**G**), 20–24 hpf (**B**,**E**,**H**), and 60–72 hpf (**C**,**F**,**I**). Expression was limited to the embryonic midline (m) at 10 hpf. At 20–24 hpf, expression was most pronounced in the notochord (n) for *Col11a1a* (chr24) and in the somites (s) for *Col11a1b* (chr2). At 60–72 hpf, developing craniofacial structures showed high levels of expression in addition to the notochord observed at 24 hpf seen for *Col11a1a* (chr24). *Col11a1b* (chr2) was also apparent in the craniofacial (cf) structures at 60–72 hpf in addition to the somites. Scale bars = 250 µm.

The spatial expression of *Col11a1a* and *Col11a1b* was determined by in situ hybridization, as shown in Figure 4. Using probes directed to exons 6a-7-8-9 for *Col11a1a* (chr 24) and exons 6-7-8-9 for *Col11a1b* (chr2), expression was detected in early zebrafish embryos. Spatial expression varied with the developmental stage. At 10 hpf, *Col11a1a* (chr24) expression was present along the dorsal midline. At 24 hpf, expression was most pronounced in the notochord and in the hindbrain. At 60–72 hpf, expression was detected in the craniofacial structures. The overall expression pattern for *Col11a1a* (chr24) was similar to that determined for *Col11a2* (chr19), as both were expressed in notochord and developing cranial cartilages [29].

*Col11a1b* (chr2) was observed in the somites at 20–24 hpf, similar to *Col5a1* [29]. *Col11a1b* (chr2) was detected within the craniofacial region at 60–72 hpf (Figure 4), similar to *Col11a1a*.

An AMO-mediated knockdown strategy was used to investigate the role of *Col11a1a* and *Col11a1b* in early development. Microinjection of 2 nL of a 0.5 mM AMO targeting the translational start site of *Col11a1a* (Col11a1a-MOe1) was lethal in 57% of treated embryos compared to 26% lethality for treatment with the AMO targeting the translational start site of *Col11a1b* (Col11a1b-MOe1) (Table 2). The knockdown of specific variants to explore the contribution of the variable region to survival was performed under the same conditions. AMOs targeting splice sites of exon 6a or exon 8 are shown in the Supplementary Materials.

Comparing the efficiency of splice-modifying AMOs indicated that the exon 6a AMO was more effective than the exon 8 AMO. Therefore, we focused primarily on the splice-altering effect of skipping exon 6a, including the AMO targeting exon 8 for comparison. Lethality levels were similar to standard morpholino control, indicating that alternative isoforms may be able to compensate for each other in the case of *Col11a1a*. The AMO targeting exon 6b, Col11a1a-MOe6b, did not affect mRNA splicing.

Notochord deformities and a shortened overall body length were observed in morphants (Table 3 and Table 4). Approximately 74% of Col11a1a-MOe1 morphants exhibited a severely curved notochord. A significantly shorter body length was observed in the remaining morphants when compared to the standard AMO control (2.81 ± 0.12 mm vs. 3.02 ± 0.17 mm; *p* = 0.0031). Col11a1b-MOe1 morphants exhibited a similar trend, with 75% having a severely curved notochord and the remaining showing a significantly shortened body length than the standard AMO control (2.67 ± 0.16 mm; *p* < 0.0001). Although AMOs directed toward splice sites of exons 6a and 8 did not alter viability, as shown in Table 2, they did have a significant effect on development and body plan. Morphants treated with the AMO preventing the inclusion of exon 6a (Col11a1a-MOe6a) showed a 93% prevalence of notochord deformity as well as a significantly shortened body length in measurable morphants (2.85 ± 0.12 mm; *p* = 0.0284). By contrast, Col11a1a-MOe8 morphants showed the lowest prevalence of notochord deformities but still demonstrated a shortened body length (2.85 ± 0.18 mm; *p* = 0.0123).

**Figure 5:**
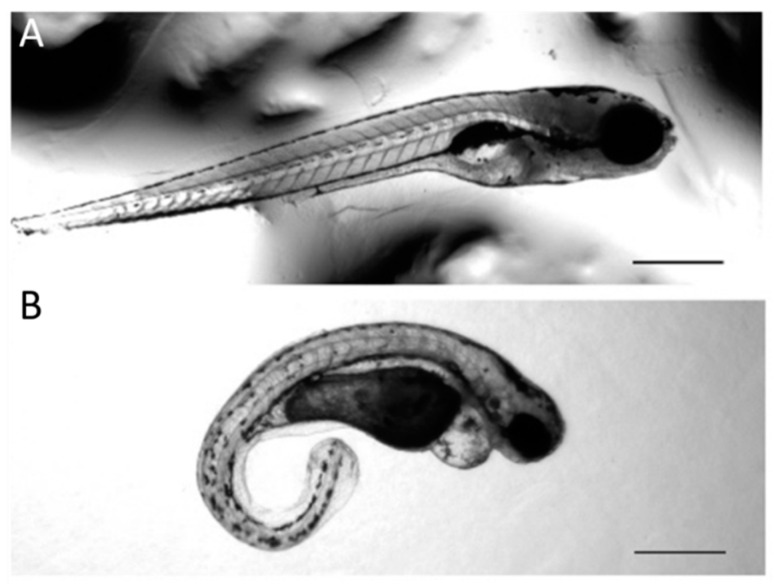
Body length and curvature changes due to Col11a1a-MOe1 AMO knockdown. (**A**) Control AMO injection observed at 72 hpf. (**B**) Col11a1a-MOe1 AMO injection observed at 72 hpf. Zebrafish embryos treated with the transcriptional start site-specific AMO for *Col11a1a* resulted in increased curvature and decreased body length. Additionally, heart edema was observed, as shown in B. Scale bar = 500 µm.

Additionally, missing or extra otoliths, pericardial edema, and smaller Meckel’s cartilage were observed in the *Col11a1a* and *Col11a1b* knockdown morphants (Table 4). To illustrate the change in body length and curvature observed due to treatment with AMOs that block protein translation by targeting the translational start site that exists with exon 1 for *Col11a1a*, representative examples are shown in Figure 5.

**Figure 6:**
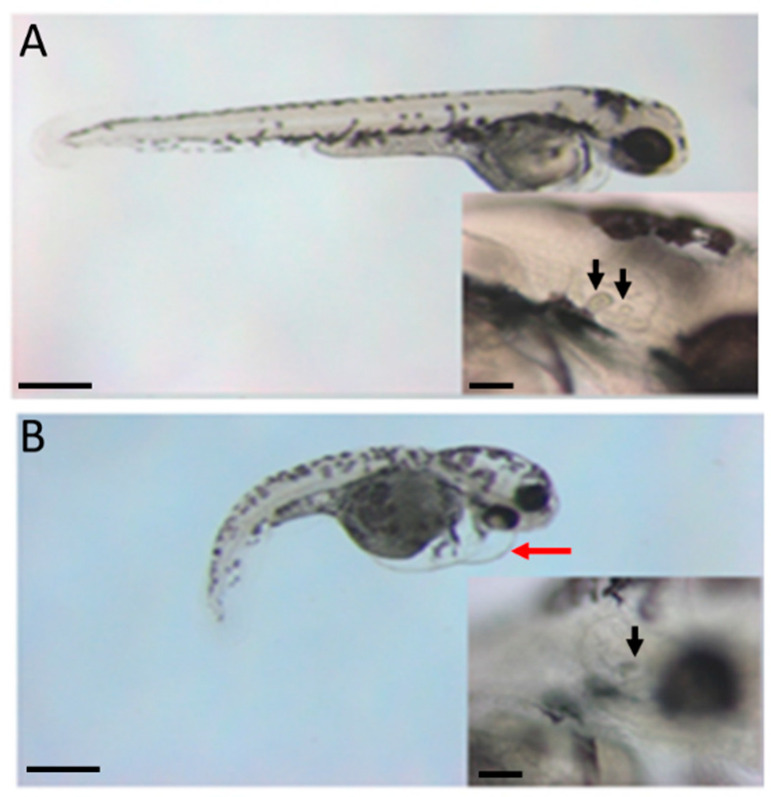
Cardiac, body length, and curvature changes due to Col11a1b-MOe1 AMO knockdown. (**A**) Control AMO injection, observed at 72 hpf. (**B**) Col11a1b-MOe1 AMO injection, observed at 72 hpf. Body length shortening, the curvature of the primary axis, and edema of the heart are apparent in zebrafish embryos treated with the transcriptional start site-specific AMO for *Col11a1b*. Otoliths are affected as indicated by arrows within the insets. Two arrows in A (inset) indicate the position of two otoliths in control zebrafish embryos. One arrow in B (inset) indicates the presence of only one otolith. Pericardial edema is indicated by the red arrow in B. Scale bar = 200 µm. Scale bars in inset represent 50 µm.

A similar phenotype was observed using the Col11a1b-MOe1, as shown in Figure 6. Treatment with the AMO that blocks protein translation by targeting the translational start site existing with exon 1 for *Col11a1b* resulted in the defects illustrated in Figure 6.

**Figure 7:**
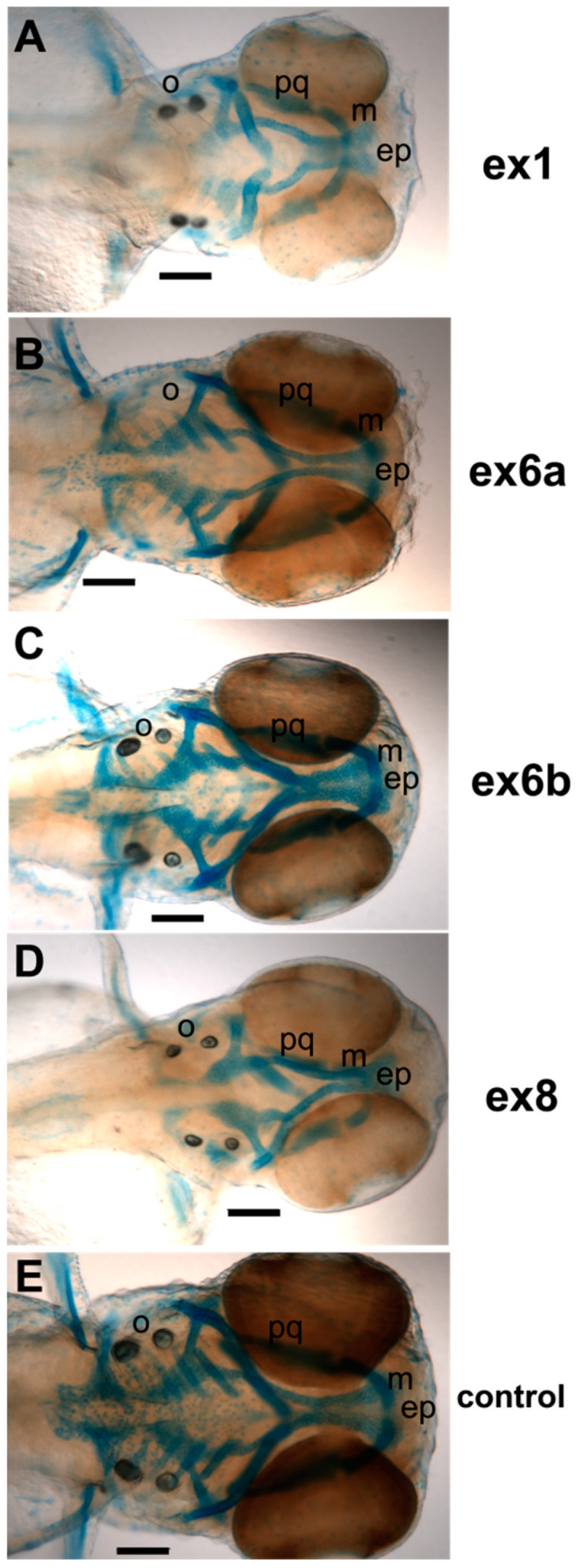
Alcian blue staining of craniofacial cartilage in 72 hpf zebrafish morphants of *Col11a1a*. A range of severity was observed among embryos of *Col11a1a* morphants. (**A**) Antisense morpholino oligonucleotide targeting the translational start site of Col11a1a-MOe1 morphants demonstrate reduced Alcian blue staining, disorganized cartilage, and shortened jaw. (**B**) Col11a1a-MOe6a morphants demonstrate reduced Alcian blue staining, shortened Meckel’s cartilage, and an absence of otoliths. (**C**) Col11a1a-MOe6b show relatively little effect and are similar to the control zebrafish. (**D**) Col11a1a-MOe8 show reduced Alcian blue staining. (**E**) Standard AMO control. Abbreviations palatoquadrate (pq); Meckel’s cartilage (m); ethmoid plate (ep); otolith (o). Scale bars = 200 µm.

We investigated *Col11a1a* in more detail, focusing on the effect of splice-modifying AMOs. A reduction in Alcian blue staining was observed in the cartilage of Col11a1a-MOe1, Col11a1a-MOe6a, and Col11a1a-MOe8 morphants (Figure 7). A reduction in the size of Meckel’s cartilage was observed. Otolith defects were observed that included a reduction in size, extra or missing otoliths.

The knockdown of genes using AMOs has certain limitations, including the duration of the AMO knockdown and the specific timing during development. Because of these limitations, we could not fully appreciate the role of specific splice forms during development. To complement the AMO approach and to increase the time frame of our investigations, we developed a model system based on CRISPR/Cas9 technology. A CRISPR/Cas9 knockout was performed, resulting in a similar phenotype to that observed by AMOs.

**Figure 8:**
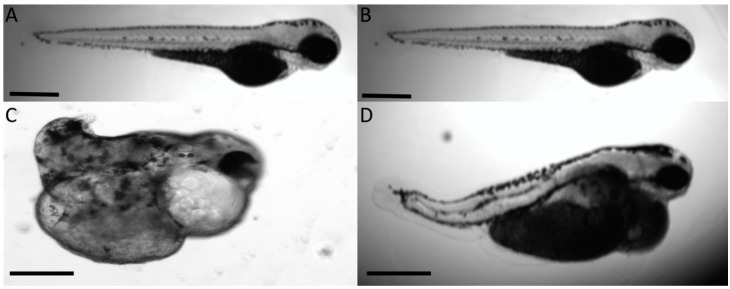
CRISPR/Cas9-mediated homozygous and heterozygous knockout of *Col11a1a* shows a similar but more severe outcome compared to AMO knockdown. (**A**) Wild-type 72 hpf embryo compared to (**C**) homozygous *Col11a1a* ^−/−^ knockout embryo at 72 hpf showing the severe effect of the complete absence of *Col11a1a* in early embryogenesis. Homozygous offspring were raised to adulthood and bred to wild type to generate heterozygous *Col11a1* ^−/+^ offspring. (**B**) Wild-type 72 hpf embryo. (**D**) Heterozygous *Col11a1a* ^−/+^ embryo at 72 hpf. Scale bar = 200 µm.

Homozygous knockout embryos displayed severe phenotypes such as that shown in Figure 8 and demonstrated a more severe lethality, as shown in Table 5. Heterozygous knockouts that resulted from crossing homozygous knockouts with a wild-type fish resulted in a lower level of lethality and a phenotype that was more similar to the AMO morphants. Representative homozygous and heterozygous offspring are shown in Figure 8.

## 4. Discussion

Mutations in the *COL11A1* gene have been identified as a cause for a range of human developmental defects resulting in facial abnormalities, eye defects, hearing loss, and articular joint defects. Zebrafish have been well established as a model for studying mammalian developmental processes and disorders resulting from genetic defects. Two chromosomal locations were investigated for *Col11a1a* and *Col11a1b* in zebrafish. Gene expression was detected throughout development. Alternative splicing was observed in the zebrafish gene *Col11a1a* but not *Col11a1b*. Knockdown of zebrafish gene expression and splice forms resulted in varying degrees abnormalities in the Meckel’s cartilage, otoliths, notochord, and heart. Additionally, shortening of total body length and embryonic lethality was observed. These data provide evidence that the zebrafish genes for *Col11a1a* and *Col11a1b* are essential for normal development and that *Col11a1a* has similar characteristics as the human *COL11A1* gene.

The development of every organ system depends on a properly organized extracellular matrix. Extracellular matrix (ECM) assembly involves the dynamic interaction around structural macromolecules as well as between cells and ECM molecules. Biosynthetic or structural deficiencies of the components of the ECM are associated with a wide spectrum of birth defects that predispose individuals to symptoms ranging in severity from mild osteoarthritis to lethal chondrodysplasia with associated eye involvement and hearing loss [49,50], designated Stickler syndrome type 2 (OMIM #604841) or Marshall syndrome (OMIM #154780) [17].

Cloning of the zebrafish orthologues *Col11a1a* and *Col11a1b* with subsequent analysis of the various splice forms presented here form the basis on which further studies of vertebrate type XI collagen function can be performed. This work also confirms the value of the zebrafish as a model for the study of the role of type XI collagen during vertebrate development.

In our studies, riboprobes localized to the structures that were affected during AMO-mediated knockdown. The zebrafish craniofacial structures are analogous to those that are affected in the *cho/cho* mice as well as the structure commonly affected by a mutation in the human *COL11A1* gene. These affected structures include a small jaw, changes to the ears that lead to hearing loss, and cleft palate [51]. In the *cho/cho* mouse, a model for chondrodysplasia, the result of a mutation in Col11a1 is dwarfism, with both chondrogenesis and endochondral ossification affected [52]. These data provide encouraging evidence that the functions of zebrafish *Col11a1a* and human *COL11A1* are conserved. If the zebrafish mutant generated in this study is an accurate model, mutants may also display cleft palate, smaller rib cage, and signs of osteoarthritis. Vison impairment and hearing loss would also be expected. Additionally, disturbed endochondral bone formation would possibly occur. Studies are now underway to investigate ear development and hearing, eye development and vision, and jaw formation with subsequent mineralization in our mutant zebrafish.

CRISPR/Cas9 gene editing was used to create a model system that expressed a lower level of *Col11a1a*. CRISPR/Cas9 has advantages over AMO knockdown because, unlike the transient effect of AMOs, the CRISPR/Cas9-mediated change is stable. Further, while AMOs are ideal for observing the effects very early in development, CRISPR/Cas9 may be useful for looking at the effect of genes that are expressed at later times in development.

Expression of the *Col11a1a* within the developing otic vesicle suggests that the zebrafish model will provide a means by which to study the further molecular and cellular basis of the hearing loss characteristic of the Marshall and Stickler syndromes.

The structural role of collagens in the formation of the ECM is well established. However, many ECM proteins also function as signaling molecules, directing the behavior of cells [53]. Interestingly, the craniofacial structures commonly affected by mutations in *Col11a1a* are derived from neural crest cells, including Meckel’s cartilage, the otic vesicle, and otoliths [7]. Perhaps a lack of proper neural crest migration or differentiation, in addition to structural abnormalities, may be responsible for the phenotypes that characterize aberrant *Col11a1a* expression [54,55]. The ECM may serve as a scaffold on which neural crest cells migrate [56], and may facilitate the onset of migration of cells of neural crest origin during emigration [57]. ECM molecules may also influence cells by inhibiting or deflecting migrating neural crest cells, thus establishing a specific developmental pattern within the developing embryo [58]. Complex patterns of alternative splicing and splice form expression may be the key to unlocking the roles played by type XI collagen, minor fibrillar collagens, and other ECM proteins.

## Figures and Tables

**Table 1:** CRISPR/Cas9 gene editing.

Name	Target	Guide Sequence ^1^	Forward Primer	Reverse Primer
E101	Exon 1	ATTTAGGTGACACTATA**GGCCAAGGTGGTCCCCAATG**GTTTTAGAGCTAGAAATAGCAAG	GGCACTTTTGGGATTGTAGAAG	CATCTCCTCTTAGAAAGCCCCT
E201	Exon 2	ATTTAGGTGACACTATA**AAGAGCATCACAGCCAGACG**GTTTTAGAGCTAGAAATAGCAAG	CTGCTGACATTTTGCATGTCTT	CATTTAAACGCAGCTGAACGTA
E301	Exon 3	ATTTAGGTGACACTATA**AGGCGTCCAGCAGCTGGGCG**GTTTTAGAGCTAGAAATAGCAAG	GTAAGAAGAAGCTGACCAAGCC	CCCGTTTATTTCTACCTCATGC
E401	Exon 4	ATTTAGGTGACACTATA**TGGCACCAGGATCCTGGATG**GTTTTAGAGCTAGAAATAGCAAG	GTAAGAAGAAGCTGACCAAGCC	CCCGTTTATTTCTACCTCATGC
E501	Exon 5	ATTTAGGTGACACTATA**GCCTGCAGTGTGTCCTTGTG**GTTTTAGAGCTAGAAATAGCAAG	GCTCTGTTTTTGGTCTCCTCAG	AGACGTCCAGAAGCGTTTAGTC
E2701	Exon 27	ATTTAGGTGACACTATA**GGTGTCCGTGGTCTAAAGGG**GTTTTAGAGCTAGAAATAGCAAG	TTCACTGTTGTCATTTTCAGGG	ACGTGTGACGATTTCTCCATTA

^1^ Bold nucleotides indicate the coding sequence with the guide sequences.

**Table 2:** Summary of lethal effect on reduced levels of *Col11a1b* and *Col11a1a* variants.

AMO	N	Lethality
Col11a1b-MOe1	54	14 (26%)
Col11a1a-MOe1	87	50 (57%)
Col11a1a-MOe6a	80	24 (30%)
Col11a1a-MOe8	56	19 (34%)
Std. AMO control	32	11 (34%)

**Table 3:** Col11a1 knockdown results in a decrease in body length.

AMO	Length (mm)	% Decrease
Col11a1b-MOe1	2.67 ± 0.16	−13.1%
Col11a1a-MOe1	2.81 ± 0.12	−7.5%
Col11a1a-MOe6a	2.85 ± 0.12	−6.0%
Col11a1a-MOe8	2.85 ± 0.18	−6.0%
Std. AMO control	3.02 ± 0.17	0

No effect was observed for Col11a1a-MOe6b.

**Table 4:** Summary of defects observed under reduced levels of *Col11a1a* and *Col11a1b.*

AMO	n	Missing or Extra Otoliths	Pericardial Edema	Curved Notochord	Smaller Meckel’s Cartilage
Col11a1b-MOe1	40	40 (100%)	39 (98%)	30 (75%)	40 (100%)
Col11a1a-MOe1	34	18 (53%)	28 (82%)	25 (74%)	33 (97%)
Col11a1a-MOe6a	44	30 (68%)	42 (95%)	41 (93%)	44 (100%)
Col11a1a-MOe8	18	0	0	1	18 (100%)
Std. AMO control	32	0	0	0	0

No effect was observed for Col11a1a-MOe6b.

**Table 5:** Summary of lethality in CRISPR/Cas9 homozygous and heterozygous mutants.

CRISPR/Cas9	N	Lethality
*Col11a1a* −/−	303	299 (98%)
*Col11a1a* +/−	299	152 (50%)
Wild-type control	311	25 (8%)

## Supplementary Materials

The following are available online at https://www.mdpi.com/2221-3759/8/3/16/s1, Supplemental Figure S1. Col11a1a-MOe6a and Col11a1a-MOe8 alter splicing pattern at 48 hpf but recover by 72 hpf. Treatment with AMOs was performed at the one- to two-cell stage and splicing was monitored at 48 and 72 hpf. Lane 1: size markers; Lane 2: PCR product amplified using primers for exon 5 and 9 of *Col11a1a* (chr 24) from 48 hpf embryos after treatment with the control AMO showing that the most prevalent splice form consists of 6a-7-8-9 at 48 hpf. Lane 3: PCR products of amplification after treatment with Col11a1a-MOe6a, showing a decrease in the most prominent splice form and the appearance of new splice forms that exclude exon 6a. Lane 4: PCR products of amplification after treatment with Col11a1a-MOe8, showing appearance of new splice forms that exclude exon 8. Lanes 5, 6, and 7: control and treatment at the 72 hpf time point demonstrate the transient effect of AMO treatment, showing that the splice patterns of the treated samples match the control. Size markers are indicated in basepairs on the left, while identity of the PCR bands is indicated on the right, referring to exons included as a result of alternative splicing. The identity of PCR products was confirmed by DNA sequencing.

## Abbreviations

Antisense morpholino oligonucleotide (AMO), hours post-fertilization (hpf), days post-fertilization (dpf), carboxyl propeptide (cpp), carboxyl telopeptide (ctp), major triple helix (MTH), minor helix (mh), amino telopeptide (ntp), amino propeptide (npp), laminin-neurexin-sex hormone binding protein (LNS), variable region (VR), kilobasepair (kbp), chondrodystrophic (cho), hours post-fertilization (hpf), days post-fertilization (dpf), chromosome (chr), pounds per square inch (psi), digoxigenin (dig), reverse transcriptase-polymerase chain reaction (RT-PCR), extracellular matrix (ECM), Online Mendelian Inheritance in Man (OMIM), and clustered regularly interspaced short palindromic repeats (CRISPR).
