# Supplementary material for: Col11a1a Expression Is Required for Zebrafish Development
Source: J Dev Biol. 2020 Aug 28;8(3):16. doi: 10.3390/jdb8030016 (PMC7558312; doi:10.3390/jdb8030016)
Supplement: Supplementary file 1 [file jdb-08-00016-s001.pdf]

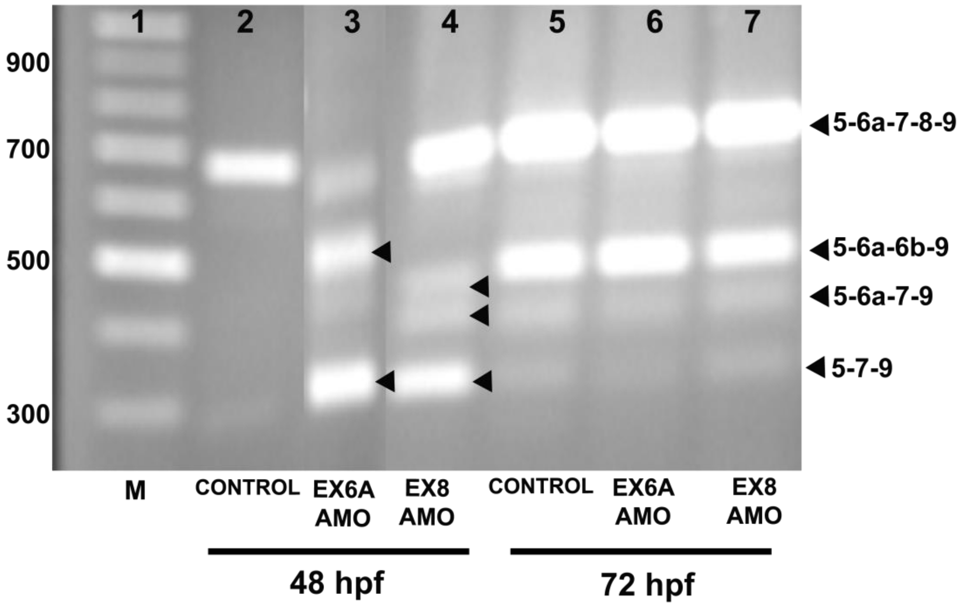

Supplemental Figure S1. Col11a1a-MOe6a and Col11a1a-MOe8 alter splicing pattern at 48 hpf but recover by 72 hpf. Treatment with AMOs was performed at the one- to two-cell stage and splicing was monitored at 48 and 72 hpf. Lane 1: size markers; Lane 2: PCR product amplified using primers for exon 5 and 9 of *Col11a1a* (chr 24) from 48 hpf embryos after treatment with the control AMO showing that the most prevalent splice form consists of 6a-7-8-9 at 48 hpf. Lane 3: PCR products of amplification after treatment with MOe6a, showing a decrease in the most prominent splice form and the appearance of new splice forms that exclude exon 6a. Lane 4: PCR products of amplification after treatment with MOe8, showing appearance of new splice forms that exclude exon 8. Lanes 5, 6, and 7: control and treatment at the 72 hpf time point demonstrate the transient effect of AMO treatment, showing that the splice patterns of the treated samples match the control. Size markers are indicated in basepairs on the left, while identity of the PCR bands is indicated on the right, referring to exons included as a result of alternative splicing.
